# The Beat

**Published:** 2007-10

**Authors:** Erin E. Dooley

## Cars, Kids, and Cigarettes Don’t Mix

**Figure:**
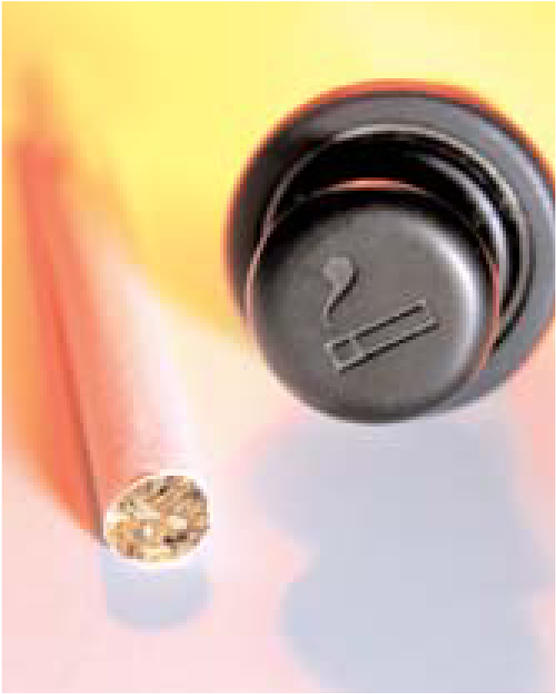


In August 2007 New York City councilman James Gennaro proposed a city ban on smoking in cars with passengers under the age of 18, following the example of similar measures in other parts of the United States and Australia. In July 2006, for example, the state of Arkansas imposed statewide restrictions on the habit. But unlike some other existing bans, Gennaro’s calls for fines of up to $2,000 for noncompliance. A study published 18 July 2007 ahead of print in the *Journal of Exposure Science and Environmental Epidemiology* noted that just two cigarettes’ worth of secondhand smoke exposes car passengers to particulates in excess of government safety standards.

## Green School Movement

Green schools, with features such as natural lighting and low-emission building materials, generally see lower rates of asthma and allergies among students and staff, better student attendance, and higher teacher retention. Green schools typically are also cheaper to operate and consume less water and energy. In July 2007, the U.S. Conference of Mayors put the weight of its 1,100-plus constituents behind a resolution calling on Congress to fund K–12 green school demonstration projects and research to better identify the payoffs of building green schools. So far, more than 30 schools have received LEED certification, with 300 more in line to do so.

## What Keeps Little Ones Up at Night

**Figure:**
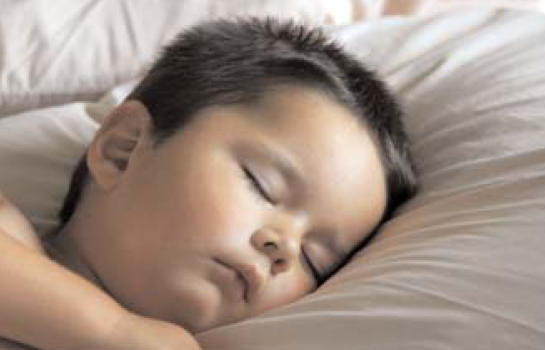


Studies in the July 2007 issue of *Early Human Development* and the September 2007 issue of *Pediatrics* have uncovered factors that may disrupt infant and toddler sleep. The first study identified a link between anxiety and depression in pregnant women and sleep problems in their offspring that can negatively affect a wide range of bodily and developmental processes. The second study found that nicotine in breast milk produces similar effects and, the authors speculate, could further increase these children’s risk of becoming smokers themselves, given that children often prefer flavors initially transmitted through breast milk.

## Lag in Label Laws

A decade ago, the FDA admitted that its regulations regarding pharmaceutical labeling needed revision to help protect pregnant women against taking products that could harm their unborn children. Although a new labeling system has been developed, it is still not in place, and the agency says implementation could still be three years away. The Public Affairs Committee of the Teratology Society published a position paper in the September 2007 issue of *Birth Defects Research Part A* calling for these changes to be implemented without delay. The paper also recommends testing the system to assess its effectiveness in conveying levels of risk and improving clinical decision making.

## Early Puberty Among U.S. Girls

The risk of breast cancer can increase by as much as 50% in women who reach menarche at age 12 compared with age 16. Other possible effects of early puberty include depression, anxiety, and eating and adjustment disorders. *The Falling Age of Puberty in U.S. Girls*, an August 2007 report published by the Breast Cancer Fund, reveals that U.S. girls are reaching puberty earlier, but the effect is greatest in overweight black girls. At age 10, three times as many black girls as white girls have begun menstruating. The onset of puberty can be affected by factors including obesity as well as pharmaceutical and other chemical exposures.

## Sussing Out High Blood Pressure in Kids

**Figure:**
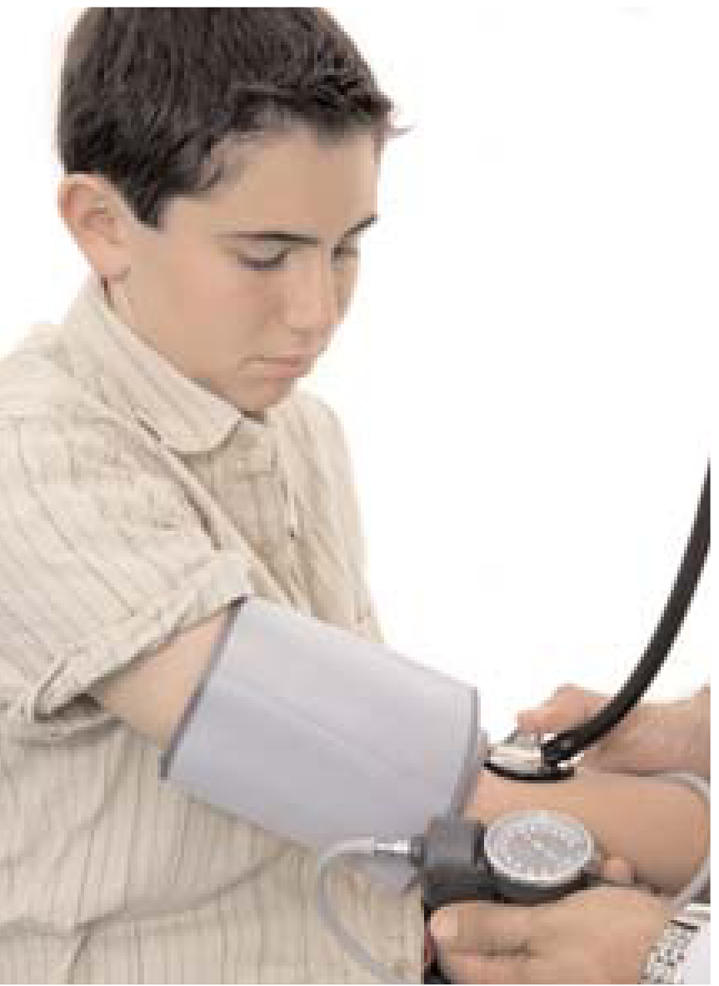


About 2 million U.S. children and adolescents are estimated to have high blood pressure, but 1.5 million of their cases may be undiagnosed, according to a study in the 22 August 2007 issue of *JAMA*. A review of more than 14,000 child medical records showed that only 26% of the children with high blood pressure as reflected in readings taken over three doctor visits had received such a diagnosis. It is not fully known how high blood pressure affects people in these age groups, although some experts speculate it may contribute to early artery and heart damage.

## Iron Deficiency in Toddlers

UT Southwestern Medical Center researchers report in the September 2007 issue of *Pediatrics* that being overweight and not attending daycare both put toddlers at high risk for iron deficiency. Iron-deficiency anemia in the formative years is linked with behavioral and cognitive delays in such areas as mental and motor development, learning, and school achievement. Hispanic toddlers were more likely than white or black children to be overweight and not in daycare, factors the authors say should be considered when implementing community-based interventions.

